# Social Media Recruitment: Communication Characteristics and Sought Gratifications

**DOI:** 10.3389/fpsyg.2019.01669

**Published:** 2019-07-16

**Authors:** Marieke Carpentier, Greet Van Hoye, Qingxiong Weng

**Affiliations:** ^1^Department of Marketing, Innovation and Organisation, Ghent University, Ghent, Belgium; ^2^The School of Management, University of Science and Technology of China, Hefei, China

**Keywords:** informativeness, organizational attractiveness, recruiting, social media, social presence, uses and gratifications

## Abstract

This study examines how social media pages can be used to influence potential applicants’ attraction. Based on the uses and gratifications theory, this study examines whether organizations can manipulate the communication characteristics informativeness and social presence on their social media page to positively affect organizational attractiveness. Moreover, we examine whether job applicants’ sought gratifications on social media influence these effects. A 2 × 2 between-subjects experimental design is used. The findings show that organizations can manipulate informativeness and social presence on their social media. The effect of manipulated informativeness on organizational attractiveness depends on the level of manipulated social presence. When social presence was high, informativeness positively affected organizational attractiveness. This positive effect was found regardless of participants’ sought utilitarian gratification. Social presence had no significant main effect on organizational attractiveness. There was some evidence that the effect of social presence differed for different levels of social gratification.

## Introduction

Employees are an important source of competitive advantage for organizations (Brymer et al., 2014). However, due to an aging workforce and an increasingly knowledge based economy in several countries across the world, competition is rising between organizations to attract and retain employees with the right skills and competencies (Ployhart et al., 2017). Accordingly, the interest in recruitment and employer branding has risen both among practitioners and scholars (e.g., Banerjee et al., 2018). Thus, research started to examine potential applicants’ reactions to selection and recruitment activities and found that their perceptions of these activities can influence their attraction toward the organization as an employer (Uggerslev et al., 2012). With regard to China, the setting of this study, research indicates that geographical and competency mismatches occur in the labor market (Peng et al., 2016; Wen et al., 2016; Athukorala and Wei, 2017). These mismatches impose difficulties for several organizations to attract the right human capital (HAYS, 2015). Due to these challenges, organizations are investing in new ways to recruit talented applicants (Lievens and Slaughter, 2016).

Both the majority of organizations and job seekers are increasingly active on social media (Nikolaou, 2014; Society for Human Resource Management [SHRM], 2016). Through these platforms organizations may influence potential applicants’ perceptions of the organization as an employer. However, there is limited knowledge about how organizations can effectively manage potential applicants’ perceptions through social media (McFarland and Ployhart, 2015). Hence, this study aims to advance the current knowledge on social media recruitment, by investigating how organizations can enhance their organizational attractiveness through the use of social media. Moreover, we aim to add to the understanding of potential applicants’ responses to social media pages in a recruitment context by investigating the impact of individual differences in terms of sought gratifications.

According to the uses and gratifications theory, people use specific media to fulfill certain needs (Katz et al., 1973). This proposition also applies to social media: people use social media to seek different gratifications (Raacke and Bonds-Raacke, 2008). In particular, utilitarian gratification and social gratification are two important motives to use social media (Azar et al., 2016; Gao and Feng, 2016; Bae, 2018). Utilitarian gratification refers to the use of social media to gather information to learn and to gain understanding of different topics. Social gratification refers to the use of social media to establish and maintain social contacts (Gao and Feng, 2016).

Guided by the uses and gratification theory, we examine two social media page communication characteristics that are conceptually related to these motives, namely informativeness and social presence (Kissel and Büttgen, 2015; Carpentier et al., 2017; Frasca and Edwards, 2017). Informativeness is the relevance and usefulness of given information for potential applicants who want to evaluate the organization as an employer (Breaugh and Starke, 2000; Van Hoye and Lievens, 2005). Social presence is the awareness of communicating with another person or entity, and has been described as the perceptions of a warm and personable communication (Short et al., 1976; Allen et al., 2013). Previous recruitment research indicates that communication characteristics can play an important role in shaping potential applicants’ perceptions (e.g., Allen et al., 2013). We now propose that actual manipulated informativeness (e.g., providing information about a day as an employee at the organization, providing information about the type of jobs) and social presence (e.g., addressing the reader directly and in a friendly way, using personal pronouns and smileys) of a social media page will have a positive effect on potential applicants’ organizational attractiveness. Additionally, we explore whether these two characteristics interact with each other in their effect on organizational attractiveness.

Furthermore, individuals can differ with regard to their sought gratifications for social media use (Orchard et al., 2014). As pages can show considerable differences in terms of content and design, sought gratifications may not always be satisfied to the same extent (Palmgreen et al., 1980). Hence, a second aim of this study is to investigate whether people’s sought gratifications on social media influence the effect communication characteristics have on potential applicants’ attraction (Gao and Feng, 2016; Bae, 2018). Investigating this can contribute to a deeper understanding of how potential applicants respond to organization’s social media use (Stoughton et al., 2015). We propose that when an organization’s social media page is more aligned with a potential applicant’s sought gratifications (in terms of communication characteristics), applicants will exhibit more positive attitudes toward the sender of that information (i.e., the organization).

In summary, this study examines (a) whether organizations can manipulate social media pages’ communication characteristics to positively affect organizational attractiveness and (b) how this effect might vary between different individuals by looking at sought gratifications for social media use. We investigate these questions by using an experimental design in which a sample of Chinese potential applicants are exposed to a fictitious company account on the social media platform WeChat.

## Social Media and Recruitment

Social media are defined as digital platforms on which users can create pages, connect with other users, generate and distribute content, and engage in interactive communication (Boyd and Ellison, 2007; McFarland and Ployhart, 2015). New social media platforms emerge, while some previously popular platforms witness a strong decrease in the number of users and some shut down. However, the number of social media users continues to grow across the globe (STATISTA, 2018). Social media allow organizations to reach out to or to find additional information on potential applicants, hence these platforms have the potential to influence the recruitment and selection functions within organizations (McFarland and Ployhart, 2015).

Many recruiters review information on job candidates on social media and use it for selection decisions (Roth et al., 2016). A study by Roulin and Levashina (2019) found that recruiters’ ratings based on candidates’ LinkedIn profiles relate to self-rated extraversion (but not to other self-rated Big-Five factors) and cognitive ability test scores. However, another study found that recruiters’ screening of a candidate’s Facebook page does not allow to predict future job performance (Van Iddekinge et al., 2016). Moreover, scholars raise some essential concerns regarding the influence of information that is irrelevant for the job and risks regarding adverse impact (Davison et al., 2016; Roth et al., 2016; Baert, 2018). Moreover, research shows that applicants’ perceptions of the selection process (e.g., the procedural justice) influence their attitudes and intentions toward that organization as a place to work (for a meta-analysis, see Hausknecht et al., 2004). Along these lines, perceptions of the use of social media as a selection tool can also influence recruitment outcomes. Indeed, initial research indicates that applicants perceive screening of the social media profile as an invasion of privacy and that this practice can result in lower organizational attractiveness and job pursuit intentions (Madera, 2012; Stoughton et al., 2015). This is thus another argument for organizations to be careful with the use of social media for the screening and selection of applicants. Overall, more research is needed investigating the use of social media for screening and selection (Davison et al., 2016).

Besides screening and selection, many organizations are also using social media to attract applicants (Society for Human Resource Management [SHRM], 2016), but in this respect research lags behind as well. Studies indicate that seeing information on social media can positively influence how potential applicants perceive an organization as an employer (Sivertzen et al., 2013; Kissel and Büttgen, 2015; Carpentier et al., 2017; Frasca and Edwards, 2017). For example, research found that self-reported exposure to information on social media was positively associated with general corporate reputation (Sivertzen et al., 2013; Kissel and Büttgen, 2015). Carpentier et al. (2017) found that exposure to an organization’s page on Facebook resulted in improved organizational attractiveness and employer brand perceptions. However, research is needed in order to better understand how organizations can effectively create and manage a social media page for recruiting purposes. To examine this, we rely on the uses and gratifications theory and investigate the role of communication characteristics of a social media page.

## Uses and Gratifications Theory and Communication Characteristics

Uses and gratifications theory roots in the communication literature (Whiting and Williams, 2013). This theory regards the audience as not being merely passive receivers of communication, but as being goal-directed in their choice of communication media. It is based on the idea that people have different motivations for using certain media. In other words, people use a specific communication medium based on certain needs that they wish to satisfy through the usage of that medium (Katz et al., 1973). While different gratifications have been studied in previous research, our study focusses on two types that have consistently been found to play a role in guiding people’s media behavior: utilitarian gratification and social gratification (e.g., Gan, 2017). Utilitarian gratification (or information seeking) is about the use of a medium to gather information or to learn. Social gratification concerns interacting and connecting with others (Whiting and Williams, 2013; Gan, 2017; Bae, 2018).

These gratifications have been found to play a vital role in social media use as well. For example, Raacke and Bonds-Raacke (2008) found that the main reason why college students used MySpace and Facebook was to keep in touch with old and current friends, but also learning about events and sharing information played a role. Leung (2013) found that social media are used to satisfy socio-psychological needs: including showing affection and fulfilling cognitive needs. A study using a Chinese sample also found that those two gratifications (utilitarian and social) were the most important motives for using Renren, a Chinese social media page (Gao and Feng, 2016).

Two communication characteristics that are conceptually related to these two motives for using social media are informativeness and social presence (Short et al., 1976; Ryan et al., 2005). These communication characteristics can thus be considered to be relevant in a social media context. We now aim to examine how social media pages affect potential applicants’ attraction by looking at these characteristics. Previous recruitment research on other recruitment channels, such as websites, found that informativeness and social presence can influence potential applicants’ reactions (Ryan et al., 2005; Allen et al., 2013). However, investigating these characteristics in a social media context remains valuable, since due to the rise of social media, information consumption has changed. Accordingly, the expectations with regard to how organizations communicate may have changed as well (Phillips-Wren et al., 2016). Examining these communication characteristics in this new context is thus useful for organizations, since we aim to provide insights into how organizations can manage their social media page in order to increase their attractiveness.

Based on signaling theory (a theory that is especially relevant in a recruitment context; Spence, 1973; Connelly et al., 2011; Uggerslev et al., 2012), job search can be considered a situation with information asymmetry. Potential applicants only have limited access to information about what the organization is like as an employer. Accordingly, they may be inclined to interpret characteristics of the organization’s communication on social media (here: informativeness and social presence) as providing signals of what the organization is like as an employer, which may influence organizational attractiveness.

Informativeness in a recruitment context is defined as the extent to which relevant, useful, and adequate information is provided for potential applicants (Van Hoye and Lievens, 2005). Based on uses and gratifications theory, gathering information is an important motive for social media use (Bae, 2018). Social media represent a new context compared to more traditional recruitment channels, for example, social media are mostly focused on relatively short messages (Papacharissi, 2009). Since information adequacy is proposed to especially play a role when little information is available, informativeness is expected to be an important factor influencing people’s perceptions in a social media context (Barber and Roehling, 1993). Consequently, whether an organization provides relevant information on their page might influence potential applicants’ attitudes toward that organization. Providing sufficient relevant information (e.g., about possible jobs and company culture) will enable potential applicants to get to know the organization as a place to work. Moreover, based on signaling theory (Spence, 1973; Connelly et al., 2011), informativeness might function as a signal of what the organization is like as an employer. An organization that is able to provide relevant and useful information to job seekers, might be perceived as more professional and competent, and hence a more attractive place to work. Along these lines, Barber and Roehling (1993) found indications that job seekers perceive job ads with little relevant information as an indicator of “sloppy, disinterested recruiting practices” (p. 853).

Of the few studies on recruitment through social media, as far as we know, two included variables related to perceived informativeness to examine how social media influence job seekers’ reactions (Kissel and Büttgen, 2015; Frasca and Edwards, 2017). Kissel and Büttgen (2015) found that the perceived available information about an organization on social media was positively related to corporate image, which in turn related positively to employer attractiveness. Frasca and Edwards (2017) found that a message on Facebook (written) and YouTube (video) was perceived as more informative than a message on the website of an organization (written). This perception was positively related to source credibility, which in turn was positively related with organizational attractiveness. These results suggest that informativeness might influence potential applicants’ attitudes toward the organization as an employer in a social media context. However, these studies only examined perceptions, rather than page characteristics. Moreover, they were not able to test causality because of their cross-sectional design. In the current study we examine whether organizations can manipulate social media page informativeness and whether this manipulated informativeness has a positive effect on organizational attractiveness. We used an experimental design to establish causality.

*Hypothesis 1.* Social media page informativeness will have a positive effect on organizational attractiveness.

Next, social presence is defined as the extent to which it feels as if you are communicating with another person. It is also described as the perception that communication is personal, friendly, and warm (Short et al., 1976). Since the maintenance and establishment of social contact is an important motive for using social media (in line with the uses and gratifications theory; Bae, 2018), the extent to which an organization communicates in a personable way may influence potential applicants’ reactions. We propose that personable communication might have a more positive influence on affect, and may result in a stronger connection with the organization. Moreover, higher social presence conveyed through a social media page may be interpreted as a signal that the organization is a friendly and warm employer, which might thus lead to improved organizational attractiveness (Connelly et al., 2011).

Allen et al. (2013) showed that perceived social presence of an organization’s website was positively associated with the attitude toward the organization. In a social media context, Carpentier et al. (2017) found that the perceived social presence of a Facebook profile was positively associated with organizational attractiveness. However, again this was a cross-sectional study which measured perceptions and could thus not test the causal relation. In the current study, we examine whether organizations can manipulate social presence on their social media page and whether it has a positive effect on organizational attractiveness.

*Hypothesis 2*. Social media page social presence will have a positive effect on organizational attractiveness.

Thus, both social presence and informativeness are proposed to increase potential applicants’ organizational attraction. Both characteristics can be manipulated independently from each other (e.g., information that is less relevant to job seekers, like product specifications, can be communicated in a personal, friendly manner). However, it is possible that the effect of informativeness on organizational attractiveness depends on the level of social presence and vice versa. On the one hand, when a page has high levels of social presence as well as informativeness, such a page may have a bigger impact on organizational attractiveness than a page that provides a feeling of friendly and personal communication, but contains no or limited relevant information or, conversely, a page that provides relevant content, but where the information is presented in an impersonal, aloof manner. We thus might propose that the social media page characteristics will strengthen each other’s effect on organizational attractiveness. On the other hand, a recruitment study by Gregory et al. (2013) found that the effect of website usability on organizational attractiveness, was higher when less job information was available. They relied on signaling theory to explain the finding (Spence, 1973): when less relevant information is available (available job information), cues that may not seem to be directly connected to the job or the organization (usability) can be used to infer what the organization is like as an employer. According to this reasoning, lower informativeness might result in an increased positive effect for social presence on organizational attractiveness and vice versa. Given that both a reinforcing and a compensating effect seem possible and there is a lack of prior research, we formulate a Research Question.

*Research Question 1*. Is there an interaction effect between informativeness and social presence on organizational attractiveness and in which direction?

In addition, as people use a social media platform with certain expectations and goals in mind (in line with the uses and gratifications theory; Bae, 2018), the extent to which these expectations are met, may influence their reactions. Individuals can have different sought gratifications for social media use in general (Orchard et al., 2014). Thus, the effect of the communication characteristics on organizational attractiveness may differ between potential applicants. We propose that potential applicants’ sought gratifications will influence the impact of social media characteristics on how they respond toward the sender of the information in terms of organizational attractiveness (Gao and Feng, 2016). Recent research indicates that the discrepancy between sought and obtained gratifications can influence satisfaction with a specific episode of social media use (Bae, 2018). Accordingly, we now zoom in on a specific social media page and propose that the extent to which an organization’s page is more aligned with a person’s sought gratifications, will relate to more positive attitudes toward the organization as an employer. In other words, the correspondence between sought gratifications and page characteristics is proposed to influence how the person perceives the organization that is sharing this communication.

Specifically, we hypothesize that the effect of informativeness on organizational attractiveness is moderated by the extent to which a person seeks utilitarian gratification. Someone who generally uses social media to obtain relevant information, may be relatively more satisfied when they encounter a page that provides useful information compared with someone who does not really use social media for information gathering purposes. A person who does not expect or aim to find much information, will be less influenced by the informativeness of the organization’s social media page.

Similarly, we expect that the effect of social presence on organizational attractiveness will be moderated by a person’s social gratification. In other words, someone who uses social media for social contact is expected to be more positively influenced by a social media page that provides a personal feeling, which will result in higher organizational attractiveness. We expect this effect to be less strong for someone who does not really use social media to satisfy social needs.

*Hypothesis 3.* The effect of informativeness on organizational attractiveness will be stronger for people with higher utilitarian gratification.
*Hypothesis 4.* The effect of social presence on organizational attractiveness will be stronger for people with higher social gratification.

To summarize, we propose that both manipulated social presence and informativeness will have a positive effect on organizational attractiveness. Moreover, we explore whether both communication characteristics interact in their effect on organizational attractiveness. With regard to the sought gratifications, we propose that the effect of informativeness on organizational attractiveness will be stronger for people with higher utilitarian gratification and that the effect of social presence will be stronger for people with higher social gratification.

## Materials and Methods

### Sample

We used a convenience sample of 200 Chinese respondents. Responses from people who took the survey multiple times (same identification number) or that showed answer biases were not included. The average age was 26 years (*SD* = 7.21) and 63% was female. Of the respondents, 55% were students (71% bachelor, 29% master) and 42% were employees (average tenure = 8.64 years; *SD* = 6.87)^1^. Both employed people and university students are relevant potential applicants as organizations might want to hire them in the close or distant future, and thus benefit from managing how they are perceived by this target population (Backhaus and Tikoo, 2004). With regard to their social media use, all respondents have WeChat, 85% indicated they follow organizations on WeChat (61% of them also reads companies’ updates). Further, when looking for work, 46% would very likely and 30% would likely look for more information about potential employers on WeChat.

### Design and Procedure

We used a 2 × 2 between-subjects experimental design to test our hypotheses. Four versions of a social media account of one fictitious company were created. The experimental variables are informativeness and social presence: the messages were manipulated to differ on the level of informativeness (high or low) and social presence (high or low). Participants randomly saw one of the conditions and subsequently assessed organizational attractiveness. To conduct a manipulation check, perceived informativeness and perceived social presence were measured next. Finally, we requested demographic information and assessed sought utilitarian and social gratifications for using WeChat.

We contacted participants by sharing a link to the online Qualtrics survey in different WeChat groups consisting of current students and alumni of a Chinese university. We also asked them to share this link with fellow students or colleagues who might be looking for jobs. Participants were rewarded with a small monetary amount after completing the survey. On the first page of the survey, instructions stated that by participating, respondents consented to the anonymous use of their responses for research purposes. We claimed that this organization had several vacancies, and that there was likely also a vacancy that fitted the profile of the respondent. Respondents were instructed to go through the provided pages of the social media account and to imagine that they wanted to find out more about the organization as a potential employer. We stressed that we wanted the participants to answer honestly as this would provide us the most valuable insights.

For the operationalization of the study, we used the Chinese platform WeChat (*Weixin*, 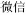). By the end of 2017, it was reported that WeChat had 989 million monthly active users (TENCENT, 2018). The platform offers a variety of features. Users can, for example, engage in real-time communication via text, voice or video messages, share photos with their contacts, and respond to information shared by contacts. WeChat plays an important role in daily life in China, many Chinese people use it to pay for their groceries, meals, or to shop online (Wang, 2018). Further, organizations can create official accounts, which users can follow to receive updates. WeChat is being used by organizations both for marketing and recruitment ends (Guillet et al., 2016).

The experimental materials were developed based on the definitions of social presence and informativeness used in this paper. The print screens (text and pictures) were developed in an iterative process in which we frequently consulted Chinese Ph.D. students. We provided the Ph.D. students the definitions of the constructs and discussed how we could improve the WeChat pages further, while keeping them as realistic as possible. The name of the fictitious company was also chosen in consultation with Chinese Ph.D. students; we chose a name that sounded neutral and realistic. To increase the realism, multiple print screens were shown as if the participant was surfing through WeChat and the interface of a smartphone was used (device most used to access WeChat). Each participant first saw a general page displaying the company name and sector, which is always seen on WeChat, before visiting an organization’s page. Next, they saw the welcome page of the fictitious organization with a short message. This message was manipulated to be either high/low on social presence and high/low on informativeness. Finally, one page was shown with two posts: one elaborated post was manipulated to be either high/low on social presence and informativeness, a second short post was added for increased realism and was kept constant across all conditions. For the low social presence condition, the messages were kept impersonal and a neutral image presenting a work desk was shown. In the high social presence condition, the messages addressed the reader directly (using the word “you”) and in a friendly way, referred to the writer as a person (“we, our” company, using a person’s name), included smileys and a picture of an employee. Recruitment-related informativeness was manipulated by either providing information on how consumers could order products (low informativeness), versus providing information relevant for job seekers, including information about the selection process, the location, and profiles of the employees (high informativeness). The study’s materials are shown in the Supplementary Appendix.

The texts for the fictitious WeChat pages were written in English and translated to Chinese in a collaborative and iterative translation process including multiple researchers (Douglas and Craig, 2007). We included as many sentences from real Chinese company’s WeChat pages and websites as possible. For the survey questions and items, the same procedure was applied and an external bureau performed a back translation. The few issues identified by the back translation were resolved together with a qualified translator (Brislin, 1970).

### Manipulation Check

We conducted a manipulation check to test the internal validity of our operationalizations. To this end, we measured perceived informativeness and perceived social presence of the social media page. The measures consisted of a combination of existing and self-developed items based on the definitions of the two constructs. For all measures and items, see Table 1. Items for perceived informativeness are based on items used by Williamson et al. (2003) and items for perceived social presence were based on Short et al. (1976) and Xu et al. (2012). All items were rated on a 7-point Likert scale (1 = *strongly disagree*, 7 = *strongly agree*). Independent sample *t*-tests using SPSS 22 showed that the perceived informativeness of the social media page was significantly higher in the high versus the low informativeness condition [*M*_high_ = 4.64, *SD*_high_ = 1.13; *M*_low_ = 3.95, *SD*_low_ = 1.33; *t*(155.61) = 3.634, *p* < 0.001]. In addition, perceived social presence was significantly higher in the high versus the low social presence condition (*M*_high_ = 4.64, *SD*_high_ = 1.29; *M*_low_ = 4.07, *SD*_low_ = 1.35; *t*(172) = 2.831, *p* = 0.005). These results confirm a successful manipulation of the communication characteristics. Moreover, these results indicate that organizations can successfully manipulate informativeness and social presence on their social media profile.

**Table 1:** Measures and Items of the Manipulation Check and Study Variables.

Variables	Items
**Manipulation check**	
Perceived informativeness	I think this page gave an accurate picture of the organization
	This WeChat account provided information that is relevant for people who are looking for a job
	This page provided detailed information about the organization as a potential employer
Perceived social presence	I felt addressed in a warm way by this account
	There was a sense of human contact in the WeChat account
	I had the feeling that I was interacting with another person
**Study variables**	
Organizational attractiveness	This organization would be a good place to work for me
	I think this organization is an attractive employer
	A job with this organization appeals to me
Utilitarian gratification	I use WeChat …
	… to get timely information
	… to get information to help me make important decisions
	… to get the information that I am interested in
	… to obtain useful information
Social gratification	I use WeChat …
	… because it allows me to get others’ opinions and advice
	… because it allows me to express my ideas
	… to see what other people say
	… to meet new people

### Measures

All items were rated on a 5-point Likert scale (1 = *strongly disagree*, 5 = *strongly agree*) and are shown in Table 1 in English (Chinese items are available on request). Internal reliabilities are displayed in Table 2.

**Table 2:** Means, Standard Deviations, Correlations, and Cronbach Alphas.

				Pearson Correlations			
	Variable name	Mean	*SD*	1.	2.	3.	4.
1.	Organizational attractiveness ^a^	3.19	0.93	(0.86)			
2.	Utilitarian gratification ^a^	3.94	0.85	0.30^∗∗^	(0.87)		
3.	Social gratification ^a^	3.58	0.91	0.40^∗∗^	0.48^∗∗^	(0.78)	
4.	Manipulated informativeness	0.51	0.50	0.31^∗∗^	0.08	0.10	
5.	Manipulated social presence	0.52	0.50	0.02	-0.09	0.08	0.02

*^a^These variables were measured on a 5-point rating scale. Manipulated informativeness and social presence are dummy variables with 0 = low level, 1 = high level. Cronbach Alphas are shown between brackets on the diagonal. ^∗∗^ p < 0.01.*

#### Organizational Attractiveness

Three items to measure attractiveness were based on previous research (Lievens et al., 2005).

#### Gratifications

Utilitarian and social gratifications were each measured with four items derived from various previous research into social media, including studies in a Chinese context. The items for utilitarian gratification come from the measures used by Lien and Cao (2014), Ha et al. (2015), and Gao and Feng (2016). The items for social gratification were used by Ha et al. (2015) and Gao and Feng (2016).

A confirmatory factor analysis was performed using MPlus 7.4. The measurement model included organizational attractiveness, utilitarian gratification, social gratification, perceived social presence, and perceived informativeness. The results indicated a good fit [χ^2^(109) = 149.558, *p* = 0.006; RMSEA = 0.043; CFI = 0.973; SRMR = 0.05]. On the contrary, a model specifying one overall factor showed a bad fit [χ^2^(119) = 716.388, *p* < 0.001; RMSEA = 0.158; CFI = 0.602; SRMR = 0.128].

## Results

Table 2 displays the means, standard deviations, internal reliabilities, and correlations of the study’s variables. Table 3 shows the mean and standard deviation of organizational attractiveness for each condition separately and shows the sample sizes and mean differences.

**Table 3:** Organizational Attractiveness per Condition: Means, Standard Deviations, Sample Sizes, and Mean Differences.

Condition	*M*	*SD*	*N*	*ΔM*^a^		
				Low SP Low Inf	Low SP High Inf	High SP Low Inf
Low SP - Low Inf	3.05	0.92	48			
Low SP - High Inf	3.30	0.86	50	0.25		
High SP - Low Inf	2.75	0.79	48	-0.30	-0.55^∗∗^	
High SP - High Inf	3.63	0.92	54	0.58^∗∗^	0.33	0.88^∗∗^

*SP = social presence, Inf = informativeness. A 1-way ANOVA indicated a significant difference in organizational attractiveness between the four conditions [F(3,196) = 9.484, p < 0.001]. ^a^ Post hoc Tukey tests were performed to examine the differences in average organizational attractiveness between pairs of conditions. ^∗^p < 0.05; ^∗∗^p < 0.01*

**FIGURE 1:**
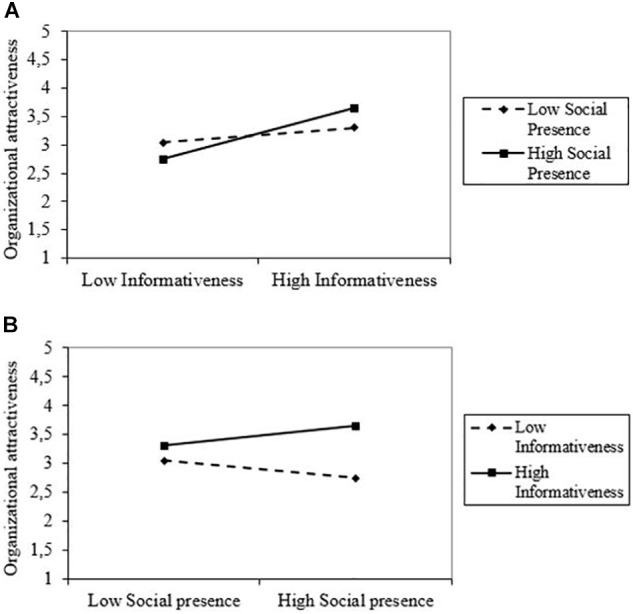
**(A)** Interaction effect of informativeness and social presence on organizational attractiveness (social presence as moderator: 0 = *low social presence*, 1 = *high social presence).* The difference in organizational attractiveness between high and low informativeness is only significant when social presence is high. **(B)** Interaction effect of informativeness and social presence on organizational attractiveness (informativeness as moderator: 0 = *low informativeness*, 1 = *high informativeness).* The difference in organizational attractiveness between low and high social presence is never significant.

Hypothesis 1 proposed a positive effect of informativeness and Hypothesis 2 proposed a positive effect of social presence on organizational attractiveness. Research Question 1 asked whether there was an interaction effect between the two characteristics with regard to organizational attractiveness. To test these hypotheses and the research question, we ran a 2-way independent ANOVA with manipulated informativeness and social presence as independent variables and organizational attractiveness as dependent variable. Organizational attractiveness was higher when manipulated informativeness was high (*M* = 3.47, *SD* = 0.9) compared to low (*M* = 2.89, *SD* = 0.87). The results of the ANOVA indicate that this difference was significant, as manipulated informativeness had a main effect on organizational attractiveness [*F*(1,196) = 20.903, *p* < 0.001, partial η2 = 0.096]. There was no significant main effect of manipulated social presence on organizational attractiveness [*F*(1,196) = 0.014, *p* = 0.907, partial η2 < 0.001]. However, the interaction of manipulated informativeness and social presence was significant [*F*(1,196) = 6.524, *p* = 0.011, partial η2 = 0.032]^2^. The interaction effect is shown graphically in Figure 1A,B.

We performed additional tests to better understand this interaction effect. Independent sample *t*-tests were performed for low and high manipulated social presence separately. Results show that when social presence was fixed to zero (low social presence condition), there was no significant effect of informativeness on organizational attractiveness [*t*(94) = -1.378, *p* = 0.172]. However, when social presence was fixed to one (high social presence condition), informativeness had a significant effect on organizational attractiveness [*t*(102) = -5.218, *p* < 0.001]. With regard to Research Question 1, these findings indicate that a high level of social presence is necessary for informativeness to have a significant positive effect on organizational attractiveness. We also tested the effect of manipulated social presence on organizational attractiveness at the different levels of manipulated informativeness using independent *t*-tests. Either when informativeness was set to zero (low informativeness condition) or when informativeness was set to one (high informativeness condition), there was only a marginal significant effect of manipulated social presence on organizational attractiveness.

Hypothesis 3 proposed that the effect of informativeness on organizational attractiveness depends on people’s level of sought utilitarian gratification. The Process macro in SPSS was used to test the interaction between manipulated informativeness and utilitarian gratification (see Table 4). 95% Confidence intervals were computed for the indirect effect based on 5,000 bootstrapped samples. Manipulated informativeness and utilitarian gratification (standardized) were entered as independent variables. Organizational attractiveness was the dependent variable. Results showed that the interaction effect was not significant. When we controlled for manipulated social presence, the interaction effect remained insignificant. Thus, no support was found for Hypothesis 3.

**Table 4:** Results of Moderation Analysis Process Macro: Interaction Informativeness and Utilitarian Gratification.

	Organizational attractiveness	
	B	95% CI
Manipulated informativeness	0.62	[0.353;0.895]
Utilitarian gratification	0.31	[0.125;0.502]
Manipulated informativeness x utilitarian gratification	0.097	[-0.282;0.475]
*R^2^*	0.198^∗∗^	

*The coefficients displayed are the unstandardized coefficients. ^∗^p < 0.05, ^∗∗^p < 0.01.*

**FIGURE 2:**
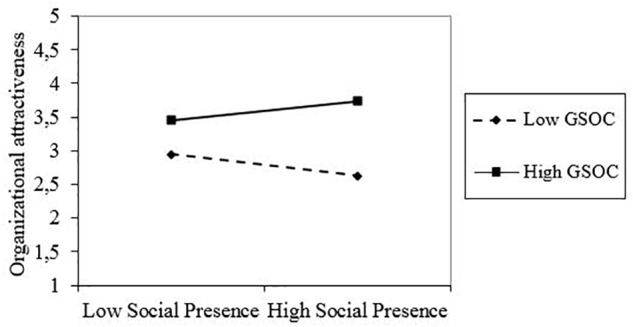
Interaction effect of manipulated social presence and social gratification on organizational attractiveness. GSOC = social gratification. The lines displayed are for the value of 1 *SD* above and below the mean of the moderator. The slope of the full line is never significant. The dotted line is only significant when the moderator (social gratification) is 1.6 *SD* below the mean. For this value: gradient of simple slope = –0.50, *t* = –1.971, *p* = 0.050 (Dawson, 2013, 2018).

Next, Hypothesis 4 proposed that the effect of social presence on organizational attractiveness depends on people’s level of sought social gratification. We again performed a moderation analysis using the Process macro (see Table 5). Manipulated social presence and social gratification (standardized) were entered as independent variables, organizational attractiveness as the dependent variable. Results show that the 95% confidence interval of the interaction term did not contain zero (B = 0.335, CI = [0.049;0.62]) When we controlled for manipulated informativeness, the interaction effect remained significant. A graph of the interaction effect is shown in Figure 2.

**Table 5:** Results of Moderation Analysis Process Macro: Interaction Social Presence and Social Gratification.

	Organizational attractiveness	
	B	95% CI
Manipulated social presence	-0.02	[-0.288;0.255]
Social gratification	0.45	[0.303;0.588]
Manipulated social presence × social gratification	0.33	[0.049;0.62]
*R^2^*	0.181^∗∗^	

*The coefficients displayed are the unstandardized coefficients. ^∗^p < 0.05, ^∗∗^p < 0.01.*

Simple slope analyses showed that the slope was only siginifcant for very low values of social gratification: below 2.12 on a scale that ranges from 1 to 5 (1.6 *SD* below the mean; only 11 respondents scored below this threshold, 151 scored higher, 38 values were missing; Dawson, 2013). For people scoring low on social gratification, a negative relation between social presence and organizational attractiveness was observed. Thus, there was some support for Hypothesis 4.

## Discussion

This study aims to improve the understanding of how organizations’ social media pages can influence potential applicants’ organizational attractiveness. Based on the uses and gratifications theory (Raacke and Bonds-Raacke, 2008), we derived two communication characteristics relevant in a social media context (i.e., informativeness and social presence). We examined whether organizations can manipulate these characteristics and whether they positively affect potential applicants’ attraction. Additionally, we investigated whether expected outcomes of social media use influence how these communication characteristics affect potential applicants’ attitudes.

This study shows that organizations can deliberately manage informativeness and social presence on their social media page. This adds to previous research, which only looked at the perceptions of these characteristics on social media (Kissel and Büttgen, 2015; Carpentier et al., 2017; Frasca and Edwards, 2017). Moreover, providing relevant information, such as the day schedule of an employee and information about company culture, improved the attitudes of potential applicants toward the organization. It might be that providing relevant recruitment related information on a social media page functions as a signal about the organization as being a good place to work (Connelly et al., 2011). These results are in line with previous research in different contexts, which showed that providing sufficient and useful information, for example on websites, in job advertisements, or in job interviews, improved applicants’ reactions (Williamson et al., 2003; Uggerslev et al., 2012; Walker and Hinojosa, 2014).

Social presence had no main effect on organizational attractiveness. However, this does not imply that communicating in a personable and friendly manner did not play a role. In fact, our findings show that informativeness only positively influenced potential applicants’ attraction, when the organization’s page also conveyed a high level of social presence. These findings thus suggest that it is important for future research to examine different communication characteristics together as their combined use can result in different reactions.

Further, as far as we know this was the first study to integrate uses and gratifications theory in recruitment research. However, this study found only limited evidence that sought gratifications on social media influence the effect of communication characteristics on organizational attractiveness. More specifically, the findings show that the positive effect of informativeness did not depend on the extent to which people use social media to obtain information. A possible explanation is the specific context of job search and recruitment. Choosing a new place to work has a great impact on people’s lives (Highhouse and Hoffman, 2001). Therefore, in this specific context, almost all people might be highly motivated to actively look for and process relevant information in order to make a well-informed choice. For social presence, there were some indications of an interaction effect, but only for a few people who indicated that they did not use social media for social purposes. For these people, high social presence had a significant negative effect on their attitudes toward the organization.

### Practical Implications

Based on the results of this study, we can provide guidelines for organizations on how to communicate on their social media pages in order to better attract applicants. First, it is important that an organization maintains its social media profiles by providing relevant information for job seekers as this is a feature that influences organizational attractiveness, independent of the reasons why individuals use social media. Organizations can, for example, provide information about the company’s current employees, its culture, the vacancies, and the selection process. Additionally, organizations should communicate this relevant information in a personable manner on their social media page. In our study, providing relevant information only resulted in positive attitudes when the information in question came across as if a friendly person was communicating it toward the reader. This can be achieved, for example, by directly addressing the reader (using personal pronouns), using pictures from employees, and including emoticons. However, results indicated that for a small subset of people this communication style made them less attracted toward the organization as a place to work. As this was the first study to investigate this, more research is necessary before further specific advice can be formulated for practice.

### Limitations and Future Research

Next, we discuss this study’s limitations and some suggestions for future research. First, we used an experimental design, which allows high internal validity, but results in uncertainty with regard to the external validity of the findings. However, a meta-analysis of recruitment outcomes by Chapman et al. (2005) indicated that differences between experimental and real applicants were small, especially in early recruitment stages in which our study is situated.

Second, we decided to use fictitious organizations’ accounts. Because of this, participants could not freely go through the WeChat account. A typical profile contains multiple pages and sometimes allows interaction through, for example, a chat robot and hyperlinks. The absence of these features might have influenced the perceived realism of the page. However, the use of fictitious accounts allowed us to keep other factors constant in order to examine causal effects.

Next, our manipulations consisted of a high and a low level of informativeness and social presence, but the difference in perceived informativeness and social presence between the high and the low conditions was not extremely large. Future research may include and compare different levels of and stronger differences in informativeness and social presence. Moreover, we performed a manipulation check to test the internal validity of our materials, however, no pilot test was performed.

The study’s main independent variables informativeness and social presence were manipulated experimentally, eliminating concerns of common method variance for these variables. However, there is still a potential issue of common method variance between the sought gratifications and organizational attractiveness. We tried to limit this possibility by first measuring organizational attractiveness, next perceived social presence and informativeness, demographics and finally the gratifications, hence trying to create more space between the measures in line with one of the recommendations by Podsakoff et al. (2003). In addition, a CFA demonstrated that a one-factor model showed a bad fit with the data.

Furthermore, caution is warranted when generalizing the findings to other contexts. Results might, for example, differ for people who feel their profile is less or more wanted by organizations. This might influence how critically they evaluate potential employers. Furthermore, future research might examine the role of communication characteristics in the different stages of the recruitment process (Breaugh, 2013). It might be that the social media page plays a stronger role early in the recruitment process, while people are still forming an initial impression of an employer. Once a person has some kind of personal experience with the organization (e.g., job interview), the information derived from this experience may weigh more strongly on their attitudes and intentions toward the organization (Cable and Turban, 2001).

Finally, this study focused on the effect of potential applicants’ exposure to information on an organization’s social media page. However, organizations can also encourage their employees to share vacancies with their personal network through social media (McFarland and Ployhart, 2015). Future research could examine which employees are more likely to act as an ambassador of their employer on social media and examine the effects of these shared messages on potential applicants’ attraction.

## Conclusion

Based on the uses and gratifications theory, this study examined how two communication characteristics of a social media page influence organizational attractiveness and whether sought gratifications moderate these effects. Findings of our experimental study show that organizations can manipulate informativeness and social presence on their social media page. Moreover, providing relevant information for job seekers on social media positively influences organizational attractiveness. However, this effect is only found when the social media page also conveys a high level of social presence. Contrary to our expectations, there is not much evidence which proves sought gratifications influence how characteristics affect potential applicants’ attitudes. Findings of this study can inspire organizations to manage their social media pages more effectively.

## Data Availability

The datasets generated for this study are available on request to the corresponding author.

## Ethics Statement

This study was carried out in accordance with the recommendations of the Research Code of Ethics, Committee Ethical Affairs, Faculty of Economics and Business Administration, UGent, with informed consent from all subjects. All subjects gave informed consent in accordance with the Declaration of Helsinki. The protocol was approved by the Committee Ethical Affairs, Faculty of Economics and Business Administration, UGent.

## Author Contributions

MC: study design, design of materials and study, data analysis, and writing of the manuscript. GVH: study design and writing of the manuscript. QW: study design, design of materials and study, and data gathering.

## Conflict of Interest Statement

The authors declare that the research was conducted in the absence of any commercial or financial relationships that could be construed as a potential conflict of interest.

## Footnotes

1 To test the robustness of our results, we performed all analyses while including a dummy variable to control for potential differences between students and employees (0 = *employee*, 1 = *student*). All main findings were similar and the dummy variable was never significantly related to organizational attractiveness, therefore we decided not to include this variable in the discussed analyses.

2 We also tested the moderation model using the Process macro in SPSS, indicating a significant moderation effect as the 95% confidence interval computed for 5,000 bootstrapped samples did not contain zero, B = 0.633, CI = [0.14;1.126].
