# Supplementary material for: Social Media Recruitment: Communication Characteristics and Sought Gratifications
Source: Front Psychol. 2019 Jul 16;10:1669. doi: 10.3389/fpsyg.2019.01669 (PMC6646858; doi:10.3389/fpsyg.2019.01669)
Supplement: Supplementary file 1 [file Table_1.DOCX]

**Appendix: Stimulus Materials**


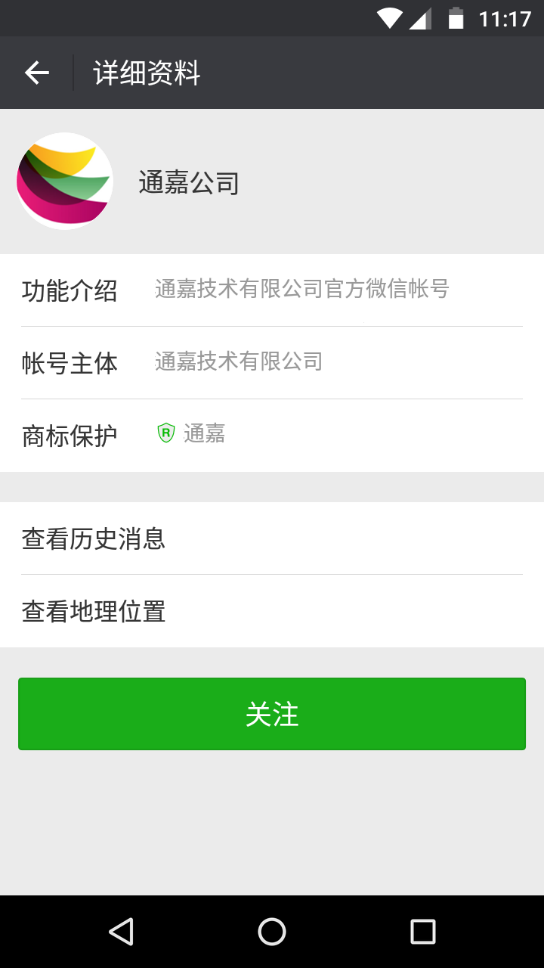
Figure A1. First page of stimulus material. The same message was shown in all four conditions. Original in Chinese, translation in English.

| **Details**  **Tongjia company**  Introduction: The official account of Tongjia technology company Account: Tongjia technology company Brand: Tongjia  View history View location  [Follow] |
| --- |

Figure A2. Second page of stimulus material: welcoming message. The Chinese print screen shown is the high social presence – high informativeness condition. The English translation of both the high and the low manipulated characteristics are displayed.

**
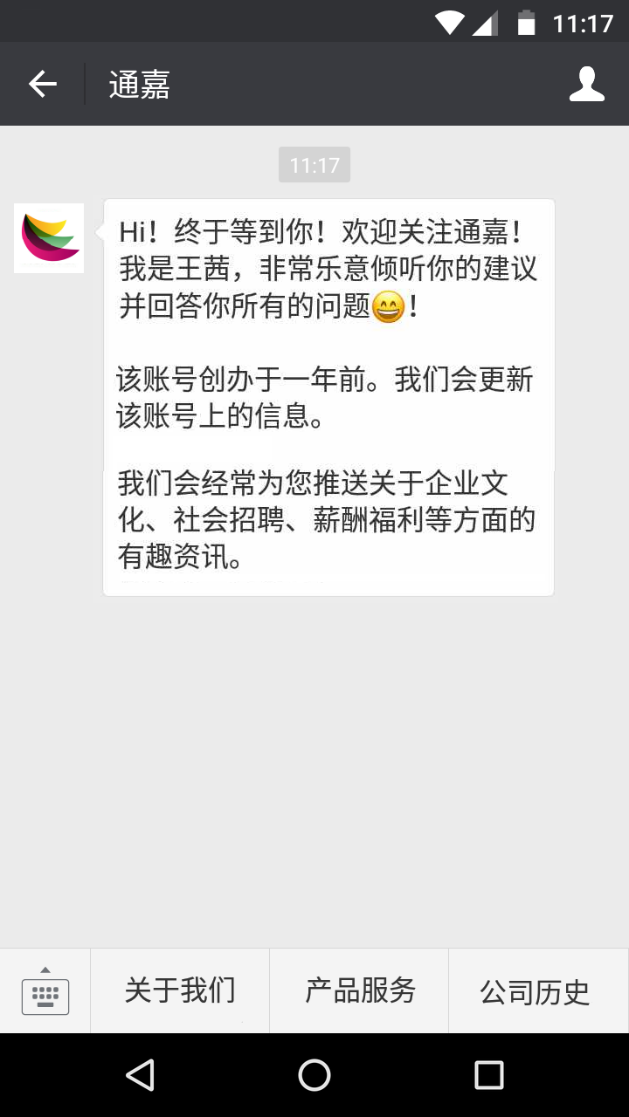
**

[**High social presence**: Hi! I was waiting for you! Welcome to the Tongjia account. I am Wang Qian and I am very willing to hear your suggestions and to answer all your questions ☺!
**Low social presence**: This is the official Tongjia WeChat account. For inquiries about product warranty, go to our sales account.]

This account is created one year ago. We post updates on this account.

[**High informativeness**: We often provide interesting information about the organization’s corporate culture, vacancies, compensation and benefits, and so on.
**Low informativeness**: Below there are three buttons: about us, product service, and company history. Click on it and a new page will open.]

About us Product service Company history

Figure A3. Third page of stimulus material: high informativeness - high social presence condition. The Chinese print screen shows two excerpts from the total page. The English translation of the manipulated message is displayed.

| 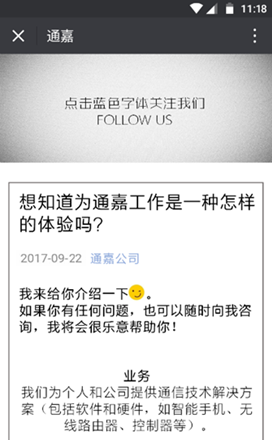  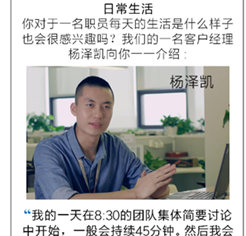 | **Do you want to know what it is like to work for Tongjia?**  I will explain it to you ☺. If you have questions afterwards, you can ask me, I’m happy to help you!  **Business** Our company provides communication technology solutions for both individuals and businesses (including software and hardware, such as smartphones, Wi-Fi transmitters, and home automation).  **Company culture** Our core values are consumers first, embrace change, and employee dedication. These values are fundamental to the way we operate, recruit, evaluate, and compensate our employees.  **Our employees** Did you know that our company has over 3500 employees from all over the country? ☺ Our employees have many different profiles, including IT, accountants, engineers, salesmen, HR**,** administrative personnel, and so on. Our organization currently has vacancies for both for recent graduates and management positions. All employees are provided the necessary training.  **Location** Our company’s headquarters are located in Shanghai and has offices in 20 cities in China, including Beijing, Chengdu, Chongqing, Fuzhou, Guangzhou, Xian, Hangzhou, Wuhan, Nanjing and so on. Possibly we also have an office in your city!  **Daily life** Are you interested in knowing what a day as an employee is like? One of our customer managers Yang Zekai explains it to you: “*My day starts at 8:30 with a team briefing to discuss current affairs which lasts about 45 minutes. Next, I go to my own office to check emails and make some calls and prepare for meetings in the afternoon. We eat lunch at 12:00 in the company canteen*.” [to read more…]  **Selection process** After applying online, you will do an online personality test and our HR manager Zhang Ruifang will call you for a telephone interview. If you pass, you will be invited for an interview at our office most close to you. Do you want to apply for a job at our organization? Sent your resume to our HR department by clicking on this link. Please explain your motivation.  Do you want to know find out more about current job vacancies, work environment, wages and benefits, advancement opportunities …? Click on this link |
| --- | --- |

| 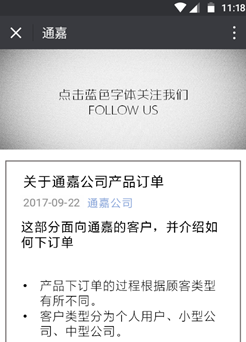**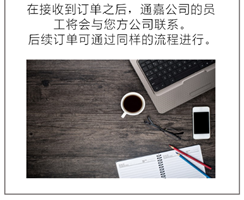** | **About ordering products with Tongjia**  This message is directed at the customers of Tongjia and introduces how to order products.   - The product order procedure differs depending on the type of customer. - There are different procedures for private persons, small sized companies, and medium sized companies   **Private persons** can buy online or in a local shop. No order form is needed.  **Small sized companies** should make use of a special order form. This form can be downloaded from our page (under the header “small companies”). The form should be filled in, signed and uploaded back on the page. For large orders, take in account that delivery times can be somewhat longer. To make sure your order arrives on time, place your order 3 days earlier.  **Medium sized companies** that order for the first time are requested to place their order through email. Email addresses of the business units can be found on the page too (under the header “departments”). Following orders can be done by the same procedure as described above. |
| --- | --- |

Figure A4. Third Page of Stimulus Material: Low Informativeness – Low Social Presence Condition. The Chinese print screen shows two excerpts from the total page. The English translation of the manipulated message is displayed.

Figure A5. Third page of stimulus material: low informativeness – high social presence condition. The print screen shows two excerpts from the total page. The English translation of the manipulated message is displayed.

| 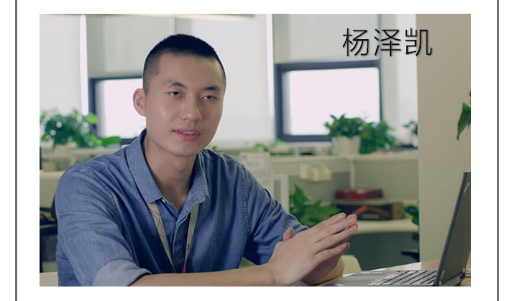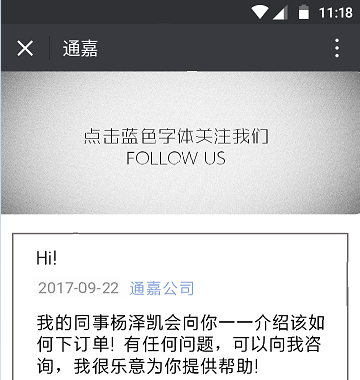 | **Hi!** Our colleague Yang Zekai will explain how you can order our products. If you have any questions afterwards, you can ask me. I would love to help you!   - There are different procedures for private persons, for small companies, and for medium-sized companies. - Our customer service manager Yang Zekai explains this below.   “Are you a private person?”  “Then you don’t need to worry about this ☺. You can just buy online or in a local shop.”  “Do you want to order for a small sized company?”  “Then you will have to use a special order form. This form can be downloaded from our page (under the header “small companies”). You have to fill in the form, sign it and upload it. For large orders, take in account that delivery times can be somewhat longer. To make sure your order arrives in time, it is best you order three days in advance.”  “Do you want to order for a medium sized company?”  “If you order for the first time, please click here to tell me which kind of products you wish to buy. I will send this to the right department. One of our employees will contact you to help you place the order.” |
| --- | --- |

Figure A5. Third page of stimulus material: high informativeness – low social presence condition. The print screen shows two excerpts from the total page. The English translation of the manipulated message is displayed.

| 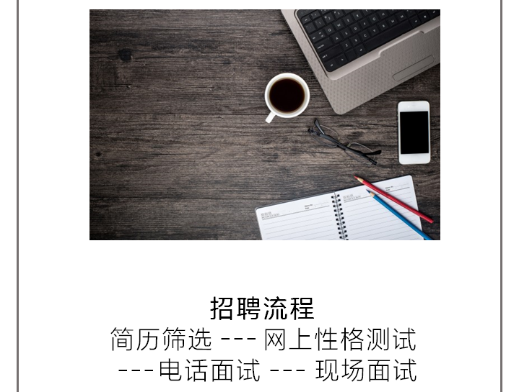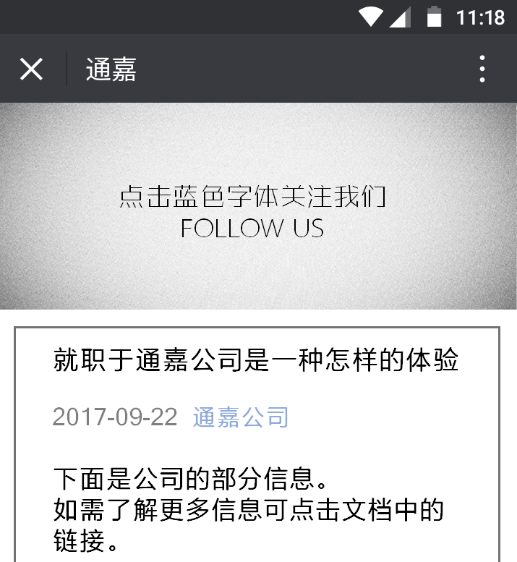 | **What it is like to work for Tongjia**  Below some information is presented about the organization. To find out more about the topics, click on the links in the texts.  **Business**  Providing communications technology solutions for both individuals and businesses (including software and hardware, such as smartphones, WIFI transmitters and home automation).  **Company culture**  The organization’s core values are customers first, embrace change, and employee dedication. These values are fundamental to the way we operate, recruit, evaluate and compensate the employees.  **Employees**  Tongjia employs over 3500 employees from all over the country. The employees have many different profiles, including IT, accountants, engineers, salesmen, HR**,** administrative personnel and so on. The company currently has vacancies for both recent graduates and management positions. All employees are provided the necessary training.  **Location**  Tongjia company has its headquarters in Shanghai. It has offices in 20 cities in China, including in Beijing, Chengdu, Chongqing, Fuzhou, Guangzhou, Xian, Hangzhou, Wuhan, Nanjing, and so on. The headquarters are located in Shanghai.  **Daily life** Below, there is a description of a day out of the life of a customer manager at Tongjia: The day usually starts at 8:30 with a team briefing to discuss current affairs, which lasts about 45 minutes. Next, people go to their own office to check emails and make some calls and prepare for meetings in the afternoon. Lunch is served at 12:00 in the company canteen. [to read more]  **Selection process** Send resume --- online personality test --- telephone interview --- face-to-face interview. To apply, please click here to send a resume to the HR department. Please explain your motivation.  Find out more about current job vacancies, the work environment, wages and benefits, advancement opportunities by clicking on this link. Frequently asked questions are also shown here. |
| --- | --- |
